# Effect of Squat Exercises on Lung Function in Elderly Women with Sarcopenia

**DOI:** 10.3390/jcm7070167

**Published:** 2018-07-05

**Authors:** Yun Kyung Jeon, Myung Jun Shin, Cheol Min Kim, Byeong-Ju Lee, Sang Hun Kim, Da Som Chae, Jong-Hwan Park, Yong Seok So, Hyuntae Park, Chang Hyung Lee, Byoung Chul Kim, Jae Hyeok Chang, Yong Beom Shin, In Joo Kim

**Affiliations:** 1 Department of Endocrinology, Internal Medicine, Pusan National University School of Medicine, Busan 49241, Korea; puritystar@hanmail.net (Y.K.J.); injkim@pusan.ac.kr (I.J.K.); 2 Medical Research Institute, Pusan National University, Busan 49241, Korea; kulu73@gmail.com (J.H.C.); yi0314@gmail.com (Y.B.S.); 3 Department of Rehabilitation Medicine, Pusan National University Hospital, Pusan National University School of Medicine, Busan 49241, Korea; lbjinishs@gmail.com (B.-J.L.); kel5504@gmail.com (S.H.K.); dasom0931@hanmail.net (D.S.C.); 4 Department of Biochemistry, Pusan National University School of Medicine, Center for Anti-Aging Industry of Pusan National University, Busan 49241, Korea; 5 Institute of Convergence Bio-Health, Dong-A University, Busan 49201, Korea; jpark@dau.ac.kr (J.-H.P.); htpark@dau.ac.kr (H.P.); 6 Department of Health Care and Science, Dong-A University, Busan 49315, Korea; seok0872@naver.com; 7 Department of Rehabilitation Medicine, Pusan National University School of Medicine, Beomeo, Mulgeum, Yangsan, Gyeongnam 50612, Korea; aarondoctor@gmail.com; 8 Department of Applied Information Technology & Engineering, Pusan National University; Busan 46241, Korea; bckim@pusan.ac.kr

**Keywords:** sarcopenia, squat exercise, respiratory function

## Abstract

We explored whether a mechanically-assisted squat exercise improved muscle mass, muscle function, and pulmonary function in elderly women with or without sarcopenia. In total, 76 community-dwelling elderly subjects (>60 years of age) were screened. We ultimately included 30 subjects who completed more than 80% of the six-week course of mechanically-assisted squat exercises (three days per week, 30 min per day). We measured body composition, lung function, knee extensor strength, hand grip strength, and the 3-min walk distance (3MWD) before and after the exercise program. Subjects with sarcopenia had poor hand grip strength and knee extensor strength, and a slow walking speed. Their lung function parameters, including forced vital capacity (FVC), was lower than those of the controls. After six weeks of squat exercises, the hand grip strength, knee extensor strength, and 3MWD increased significantly in both groups. Appendicular skeletal muscle mass and leg lean mass were increased in subjects without sarcopenia. The FVC (L) increased significantly only in the sarcopenia group (*p* = 0.019). The mechanically-assisted squat exercise program increased muscle function and lung function, including FVC, in patients with sarcopenia. Muscle mass increased in subjects without sarcopenia.

## 1. Introduction

Societies are aging worldwide. Thus, geriatric syndromes, including sarcopenia, affect the elderly both economically and physically [1]. Twenty-five years ago, the term “sarcopenia” was coined by Rosenberg [2] as a descriptor of age-related loss of muscle mass and function. Sarcopenia was associated with poor health status and adverse outcomes in terms of morbidity and mortality [3,4]. In addition, sarcopenia was reported to affect pulmonary function [5]. Several causes of sarcopenia have been reported, including the loss of motor neurons, age-related decreases in sex hormone levels, inadequate nutrition, and immobilization [6]. One important mechanism is reduced physical activity and lack of exercise. Therefore, exercise may be useful to prevent the development of sarcopenia. Exercise, especially high-intensity exercise, may improve muscle strength [7] and muscle mass [8,9], but its efficacy varies according to the type and intensity of the exercise [10]. The effects of exercise on pulmonary function have also been reported to differ among subjects and by type of exercise [11,12]. However, few studies have explored changes in respiratory function after exercise in subjects with sarcopenia. The squat exercise is a functional movement performed by flexing and extending the hip, knee, and ankle joints. It activates a wide range of supporting muscles of the trunk, including the quadriceps, hamstrings, and gastrocnemius [13], thus improving functional performance and facilitating postural stabilization of the trunk [14].

In this study, we investigated whether a mechanically-assisted squat exercise could increase muscle function and mass, and improve lung function, in community-dwelling elderly individuals with and without sarcopenia.

## 2. Methods

### 2.1. Study Design

The study protocol was approved by the institutional review board (approval number: 1504-015-029) of Pusan National University Hospital, Busan, Korea. All subjects gave written informed consent and were evaluated by physicians to ensure that they were free of all significant pulmonary, cardiac, neurological, and other diseases that could affect the study results. The study was registered in the UMIN Clinical Trials Registry (No. UMIN000020650). We performed subgroup analysis of randomized clinical trial data to explore the effects of a mechanically-assisted squat exercise (AMF-101, Foretek Microsystem, Seoul, Korea) on patients with or without sarcopenia.

### 2.2. Subjects

All participants were recruited via posters or regional health center websites. In total, 76 community-dwelling elderly subjects (aged >60 years) were screened. We prospectively included 47 such patients, who were randomly assigned to the exercise or control group in a 2:1 ratio. The exclusion criteria were: (i) kidney disease or a serum creatinine level of >1.2 mg/dL, (ii) liver disease or a serum AST or ALT level of >60 IU/L, (iii) uncontrolled diabetes mellitus (a fasting blood glucose level of >125 mg/dL), (iv) uncontrolled hypertension or a cardiac disease such as angina pectoris or myocardial infarction, (v) participation in another clinical trial during the prior 4 weeks, (vi) steroid or hormonal therapy that could affect the results, and (vii) any other reason for exclusion considered appropriate by the researchers. All subjects were non-smokers. Ultimately, we included 30 subjects who completed more than 80% of the 6-week course of mechanically-assisted squat exercises (3 days per week, 30 min per day); 10 patients with sarcopenia and 20 healthy non-sarcopenic women were analyzed.

### 2.3. Pulmonary Function Tests

A MicroLab ML3500 MK8 platform (CareFusion; Becton, Dickinson and Company, Basingstoke, Hampshire, UK) was used to perform the pulmonary function tests; an experienced doctor ran all tests [15]. All evaluations were performed with the subjects seated, as suggested by the American Thoracic Society and European Respiratory Society standards [16]. We measured the forced vital capacity (FVC), forced expiratory volume in 1 s (FEV1), maximal inspiratory pressure (MIP), and maximal expiratory pressure (MEP).

### 2.4. Knee Extensor Strength

A handheld dynamometer (HDD) (JTECH Medical, Salt Lake City, UT, USA) was used to evaluate quadriceps strength; the reliability of this method has been validated [17]. Briefly, with each subject sitting, the knee joint was flexed from the maximum extension angle by 35°, after which an HHD was positioned and fixed on the ankle to allow for isokinetic dynamometry recording of the isometric peak flexion forces (the *N* values) of both knee joints. The HDD was fixed two finger breadths above the ankle joint and distal to the tibia, using a belt. Fixed HHDs were placed at the lower edges of the slanted surfaces of slanting boxes using a belt. Each slanted surface was firmly fixed to the bottom of the box during testing, preventing any movement or lift [17]. Each flexion position was chosen by reference to the high-level surface electromyographic data yielded when the knee joint was flexed by 35° [18], and the reliability of the test data obtained when the knee joint was flexed by 35° in the HHD test was confirmed by measuring knee extensor torque [17].

### 2.5. Grip Strength

Hand grip strength (HGS) was assessed using a hand-held digital grip dynamometer (T.K.K 5401 Grip D, Takei Scientific Instruments, Tokyo, Japan). In a standing position with their arms hanging normally beside the body and the elbow angle at approximately 180°, the participants were asked to squeeze the dynamometer as hard as possible to measure the maximum force for each hand. Two measurements (in kg) for each hand were recorded, and the mean of the four measurements for each participant (M, SD) was calculated and used in the analysis [19].

### 2.6. Three-Minute Walk Test

The 3-min walk distance (3MWD) evaluates the maximum distance that can be covered along a 30 m-long corridor during a 3 min period. Two plastic cones delimited the corridor, and 2 m distance intervals were indicated by pieces of tape. Participants were instructed to walk along the walkway as fast as possible and to stop when needed. The assessor walked alongside the participants to ensure their safety and provided standardized verbal encouragement at one and two minutes: “you are doing well” and “keep up the good work”. The test ended at the end of the 3-min period, and it was stopped immediately if the participant reported chest pain, dizziness, or dyspnea. The total distance covered (in meters) was recorded [20].

### 2.7. Whole-Body Bone and Lean Mass

Dual-energy X-ray absorptiometry data were recorded using a bone densitometer (Lunar; GE Medical Systems, Madison, WI, US). A cerium filter, which acts as an electronic K-shell absorption edge, was used with a tube voltage of 80 kV to produce two intensity peaks with energies of 40 and 70 keV. The absorptiometry findings were quantified by measuring the tissue absorptions of photons emitted at two energy levels; this resolves the bodily components into bone mineral, and lean and fat soft tissue masses. The appendicular skeletal muscle mass (ASM) was calculated as the sum of the muscle masses of the arms and legs. Low muscle mass was defined as an ASM divided by body weight (ASM/Wt, %) that was one or two standard deviations (grade I or II sarcopenia, respectively) below the sex-specific mean of a young reference group [21,22].

### 2.8. Definition of Sarcopenia

We used the consensus definition of the Asian Working Group for sarcopenia [21,22,23]. All subjects were aged 60 years or older and lived in the community. When the handgrip strength and/or gait speed is low, muscle mass measurement is recommended to diagnose sarcopenia. No cutoff value of grip strength has been defined for Korean subjects; we used the cutoff employed for Japanese. This was 19.3 kg based on that of the lowest quartile of Japanese community-dwelling elderly women [24]. The Asian Working Group on sarcopenia defined a gait speed of 0.8 m/s as the cutoff reflecting low physical performance [24]. The cutoff values of muscle mass were set at one and two standard deviations below that of young adults. Kim et al. [22] defined a muscle mass cutoff of 32.2% for women and 25.6% for men, calculated as follows: ASM/body weight (%). The cited authors used data from the Fourth Korean National Health and Nutritional Examination Survey.

### 2.9. Intervention

**Figure 1:**
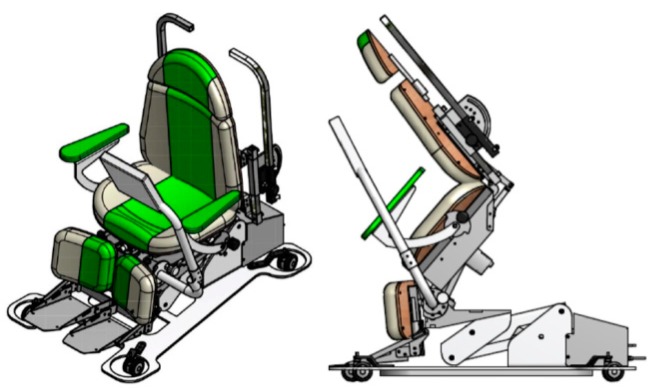
The mechanically-assisted squat exercise medical device.

A structured program of squat exercises was conducted for 30 min, three times per week on alternate days, for 6 weeks at the hospital. The mechanically-assisted squat device program was as follows: Sit down—supine—tilt—squat. The program was a recursive exercise. All participants bent the hips and knees as much as possible while straightening the back (the squat position). When participants felt fatigue or discomfort in the legs, they could lean on the machine or change their position from squat to tilt or sit, aided by a mechanical device (Figure 1). The participants were asked to score their exercise intensity during each session using the Borg scale [25]. Participants performed the exercise program for 30 min at a rating of perceived exertion (RPE) of 12–14 during weeks 1–3. After week 3, the emphasis was placed on reaching and maintaining exercise intensity at RPE of approximately 14–16 for 30 min (AMF-101; Foretek Microsystem Co., Ltd., Seoul, Korea).

Each participant was asked to assess RPE on the squat machine for 3–4 min to determine his or her squat position for the main trial. The squat position was defined as feeling between somewhat difficult and difficult (i.e., RPE of 12–16 while in the squat position). Subsequently, each participant changed position from sitting to supine to tilt, with 1–2 min rest periods. They performed 6–7 rotations of these positions, and the exercise intensity was increased slightly (i.e., the squat position time was delayed). All exercise sessions were supervised by an experienced trainer.

### 2.10. Blood Sampling

All study participants in both the mechanically-assisted squat and control groups underwent clinical evaluation and gave a blood sample after 8 h of fasting before commencement of the 6-week intervention program.

### 2.11. Statistics

All study participants in both the mechanically-assisted squat and control groups underwent clinical evaluations and gave a blood sample after an 8 h fast before commencing the 6-week intervention program. All statistical analyses were performed with SPSS for Windows ver. 17.0 (SPSS, Inc, Chicago, IL, USA). The baseline characteristics of the study groups were compared using independent Student’s *t*-tests for continuous variables. The paired *t*-test was used to compare the parameters before and after 6 weeks according to the existence of sarcopenia in both the exercise and non-exercise groups. Results were considered to be significant when the *p*-value was less than 0.05.

## 3. Results

The mean age of the study subjects was 73.8 ± 5.9 years, and the mean body mass index (BMI) was 25.2 ± 3.7 kg/m^2^. The baseline clinical and biochemical characteristics of all subjects are shown in Table 1 by the presence of sarcopenia. The patients without sarcopenia were significantly taller than those with sarcopenia (*p* = 0.005). The mean weight, BMI, and weight circumference were not significantly different between the two groups. Muscle mass estimates for the arm and leg were significantly lower in subjects with versus without sarcopenia (*p* = 0.045, and *p* = 0.012, respectively). The ASM was also lower in the sarcopenic group (*p* = 0.013). Muscle function, estimated by hand grip strength on both sides, was stronger in subjects without sarcopenia (*p* = 0.017 for the left, *p* < 0.001 for the right). The difference in HSG was more dramatic for the right arm, possibly because most subjects were right-handed. The knee extensor strength showed a trend towards being higher in subjects without sarcopenia but was not significantly different between sides. Physical performance, in terms of walking speed, was significantly better in the non-sarcopenic group (*p* = 0.024). Regarding respiratory function, subjects with sarcopenia showed a trend towards having a lower FEV1 (L) (1.57 ± 0.34 vs. 1.83 ± 0.32, *p* = 0.053) and a lower FVC (L) (1.71 ± 0.38 vs. 2.12 ± 0.34, *p* = 0.006).

After six weeks of mechanically-assisted squat exercises, weight was increased in the non-sarcopenic group (*p* = 0.001). Muscle mass, estimated by leg lean mass and ASM, was significantly increased in subjects without sarcopenia (*p* = 0.037 for leg lean mass, *p* = 0.013 for ASM) but not in those with sarcopenia (*p* = 0.059 for leg lean mass, *p* = 0.114 for ASM).

The knee extensor muscle strength and hand grip strength, representing muscle function, also increased significantly after six weeks of exercise in both the sarcopenic and non-sarcopenic subjects (*p* = 0.005 and *p* = 0.001, for knee extensor muscle strength, and *p* = 0.005 and *p* < 0.001 for hand grip strength, respectively). Physical performance, indexed by gait speed, was improved in both the sarcopenic and non-sarcopenic subjects (*p* = 0.005 and *p* < 0.001, respectively). Regarding lung function, both the FVC (L) and FVC (%) were significantly increased in subjects with sarcopenia (*p* = 0.019 and *p* = 0.041, respectively), but not in those without sarcopenia (*p* = 0.627 and *p* = 0.210, respectively) (Table 2).

## 4. Discussion

This study demonstrated that mechanically-assisted squat exercises improved muscle function, including the strength of both knee extension and hand grip, in subjects with and without sarcopenia. The muscle mass estimated by leg lean mass and ASM was increased in subjects without sarcopenia. The squat exercise improved respiratory function, as indexed by FVC, in community-dwelling elderly women with sarcopenia.

The squat is a very popular strengthening exercise; it trains primarily the quadriceps, hamstring, and gluteus muscles and strengthens bone. It also improves the function of the core muscles, balance, and gait ability. However, the squat exercise could be difficult for some elderly people; therefore, we devised a novel, mechanically-assisted squat device. Muscle function, indexed by grip strength and extensor muscle strength, was significantly increased after the six-week exercise program in subjects with and without sarcopenia. This result is consistent with previous studies that showed a positive effect of exercise on muscle function [26]. Based on these results, exercise may be considered to improve muscle function, regardless of the baseline muscle mass or muscle function. Regarding muscle mass, the six-week squat exercise program increased the ASM and leg lean muscle mass only in subjects without sarcopenia. Moreover, BMI was increased after both groups are due to the increase in ASM.

Although there was improvement in muscle function in those with sarcopenia, the muscle mass did not change significantly. Muscle function may be more sensitive to mechanical stimuli compared with muscle mass, although the exact mechanism of this dissociation is not known. The significant increase in the FVC is difficult to explain but is probably due to the lack of sufficient training intensity or duration to improve the intercostal muscle volume in subjects who already had a reduced muscle mass and function. In other words, it may be necessary to consider increasing the intensity and duration of exercise in patients with sarcopenia, as compared to those without sarcopenia. We hypothesize that the improved pulmonary function in sarcopenia patients is due to the increase in daily activity, as the squat movement also improved walking ability.

A previous study showed that low pulmonary function was related to a high risk of sarcopenia in community-dwelling older adults [27], and fat-free mass in the trunk and mid-thigh muscle mass was positively associated with lung function [28]. However, few prospective studies have evaluated the efficacy of exercise for improving lung function in subjects with sarcopenia. This study shows that baseline lung function is reduced in subjects with sarcopenia, and that a mechanically-assisted squat exercise can improve FVC in sarcopenia patients. A similar study reported an increase in FVC after exercise in young, sedentary Iranian women [29]. The authors suggested that an increase in FVC after exercise may be associated with the high rate of ventilation that occurs during exercise, or could be a secondary outcome of improved functional capacity, as suggested by Aagaard et al. [30]. Subjects with sarcopenia need aerobic exercise, but they also need resistance exercises to increase muscle mass and strength. To do this, it is necessary to train the most representative large muscle in the body (e.g., the quadriceps, hamstrings, etc.). In addition to the above-mentioned advantages, the squat movement is performed in a static posture, so the risk of injury is low. In patients with sarcopenia, it is encouraging that squat monoculture improves both muscle and respiratory function.

This study had several limitations. First, we did not limit our study to men at first. Ten out of 47 subjects were men, who were excluded for this subgroup analysis. We also excluded the subjects who did not achieve at least 80% of the exercise program. As the machine was a newly attempted method, all exercise interventions were performed in the hospital for safety. There were seven people who could not complete the program process for personal reasons, but none had any pain or other medical problems during the exercise program. These efforts reduced subject heterogeneity, but the number of subjects decreased as a result. Second, protein is an important factor in the pathophysiology of sarcopenia. It is known that dietary protein intake, and the resulting increased availability of plasma amino acids, stimulates muscle protein synthesis [31]. Therefore, protein intake plays an integral part in muscle health. Protein, as well as nutritional status such as acid-base balance, vitamin D/calcium, and other minor nutrients, could impact on muscle function and mass. Unfortunately, we did not include nutritional intake data. Third, pulmonary function was not a primary study endpoint. Nevertheless, this is the first prospective work to evaluate the effects of mechanically-assisted squat exercises on muscle mass, muscle function, and lung function in subjects with sarcopenia.

## 5. Conclusions

The efficacy of exercise may differ depending on the type and intensity of the exercise, and by the characteristics of subjects, such as the presence of sarcopenia. A prospective randomized trial exploring the effects of mechanically-assisted squat exercise by subjects with sarcopenia is essential to definitively confirm the efficacy of such exercise.

## Figures and Tables

**Table 1:** Baseline characteristics of subjects with (*n* = 10) and without (*n* = 20) sarcopenia.

Parameter	With Sarcopenia	Without Sarcopenia	*p*-Value
Age (years)	75.4 ± 5.3	73.0 ± 6.2	0.302
Height (cm)	149.0 ± 4.4	153.6±3.7	0.005 *
Weight (kg)	55.9 ± 6.4	58.1 ± 6.9	0.444
WC (cm)	85.4 ± 7.6	82.3 ± 7.0	0.279
BMI (kg/cm^2^)	25.3 ± 3.2	24.7± 3.1	0.651
ASM (kg)	11.8 ± 1.1	13.4 ± 1.8	0.013 *
Arm lean mass (kg)	3.2 ± 0.5	3.7 ± 0.6	0.045 *
Leg lean mass (kg)	9.4 ± 0.9	10.7 ± 1.4	0.012 *
Left HGS (kg)	18.1 ± 3.4	21.8 ± 4.0	0.017 *
Right HGS (kg)	16.9 ± 1.8	22.7 ± 3.3	<0.001 *
Left knee extensor (Nm)	121.5 ± 34.9	135.2 ± 26.2	0.239
Right knee extensor (Nm)	114.2 ± 41.5	144.0 ± 35.7	0.051
3MWD (m)	226.8 ± 46.8	261.0 ± 34.2	0.024 *
AST (mg/dL)	19.5 ± 4.7	22.7 ± 6.5	0.247
ALT (mg/dL)	26.8 ± 32.1	17.4 ± 6.3	0.385
BUN (mg/dL)	16.7 ± 4.1	16.9 ± 4.0	0.867
Cr (mg/dL)	0.73 ± 0.18	0.73 ± 0.16	0.780
TC (mg/dL)	192.3 ± 47.3	189.0 ± 45.0	0.854
HDL-C (mg/dL)	51.5 ± 20.1	59.3 ± 13.1	0.658
LDL-C (mg/dL)	106.4 ± 48.1	116.0 ± 37.8	0.552
TG (mg/dL)	188.4 ± 171.9	110.0 ± 54.4	0.190
FFA (mg/dL)	569.7 ± 292.9	660.4 ± 246.6	0.380
25(OH)D (ng/mL)	42.3 ± 10.2	48.9 ± 19.4	0.325
FEV1 (L)	1.57 ± 0.34	1.83 ± 0.32	0.053
FEV1 (% pred)	106.7 ± 26.5	102.3 ± 29.3	0.696
FVC (L)	1.71 ± 0.38	2.12 ± 0.34	0.006 *
FVC (% pred)	89.1 ± 22.4	94.0 ± 21.8	0.574
FEV1/FVC	0.86 ± 0.09	0.89 ± 0.06	0.230
MEP (cmH_2_O)	50.3 ± 18.8	48.4 ± 13.5	0.753
MIP (cmH_2_O)	50.3 ± 17.1	52.9 ± 15.4	0.684

WC: waist circumference, BMI: body mass index, ASM: appendicular muscle mass, HGS: hand grip strength, 3MWD:3-min walk distance, AST: aspartate transaminase, ALT: alanine transaminase, BUN: blood urea nitrogen, Cr: creatinine, TC: total cholesterol, HDL-C: high-density lipoprotein cholesterol, LDL-C: low-density lipoprotein cholesterol, TG: triglycerides, FFA: free fatty acids, 25(OH)D: 25-hydroxyvitamin D, VC: vital capacity, FEV1: forced expiratory volume in 1 s, FVC: functional vital capacity, MEP: maximal expiratory pressure, MIP: maximal inspiratory pressure. * *p* < 0.05.

**Table 2:** Comparing effects of the exercise program according to sarcopenia status; changes in anthropometric measurements, muscle strength, and pulmonary function before and after the six-week mechanically-assisted squat exercise program.

	With Sarcopenia (*n* = 10)			Without Sarcopenia (*n* = 20)		
	Before	After	*p*-Value	Before	After	*p*-Value
Weight (kg)	55.9 ± 6.4	56.7 ± 6.7	0.059	58.1 ± 6.9	58.7 ± 6.9	0.001 *
Height (m^2^)	149.0 ± 4.4	148.8 ± 4.2	0.089	153.6±3.7	153.7 ± 4.3	0.798
WC (cm)	85.4 ± 7.6	87.9 ± 7.8	0.333	82.3 ± 7.0	83.5 ± 7.9	0.205
ASM (kg)	11.8 ± 1.1	12.1 ± 1.0	0.114	13.4 ± 1.8	13.7 ± 1.7	0.013 *
Arm lean mass (kg)	3.2 ± 0.5	3.3 ± 0.5	0.445	3.7 ± 0.6	3.7 ± 0.6	0.211
Leg lean mass (kg)	9.4 ± 0.9	9.6 ± 0.8	0.059	10.7 ± 1.4	10.9 ± 1.4	0.037 *
Left HGS (kg)	18.1 ± 3.4	19.1 ± 3.3	0.005 *	21.8 ± 4.0	23.3 ± 4.0	<0.001 *
Right HGS (kg)	16.9 ± 1.8	19.3 ± 2.6	0.005 *	22.7 ± 3.3	24.7 ± 3.8	<0.001 *
Left knee extensor (Nm)	121.5 ± 34.9	170.7 ± 41.4	0.005 *	135.2 ± 26.2	195.4 ± 52.9	<0.001 *
Right knee extensor (Nm)	114.2 ± 41.5	158.3 ± 48.9	0.005 *	144.0 ± 35.7	203.3 ± 47.6	<0.001 *
3MWD (m)	226.3 ± 47.2	241.1 ± 39.2	0.005 *	261.7 ± 33.3	275.2 ± 33.7	<0.001 *
TC (mg/dL)	192.3 ± 47.3	183.7 ± 48.7	0.721	189.0 ± 45.0	180.0 ± 42.0	0.014
HDL-C (mg/dL)	51.5 ± 20.1	49.5 ± 17.3	0.959	59.3 ± 13.1	58.4 ± 13.8	0.468
LDL-C (mg/dL)	106.4 ± 48.1	106.9 ± 50.1	0.959	116.1 ± 37.8	111.9 ± 35.4	0.285
TG (mg/dL)	188.4 ± 171.9	161.9 ± 83.8	1.000	110.0 ± 54.4	106.4 ± 34.5	0.940
FFA (mg/dL)	569.7 ± 292.9	532.9 ±168.6	0.646	660.4 ± 246.6	691.9 ± 325.9	0.911
25(OH)D (ng/mL)	42.3 ± 10.2	37.8 ± 11.7	0.241	48.9 ± 19.4	47.8 ± 21.7	0.968
FEV1 (L)	1.57 ± 0.34	1.49 ± 0.42	0.139	1.83 ± 0.32	1.88 ± 0.29	0.390
FEV1 (%)	106.7 ± 26.5	95.7 ± 29.6	0.139	102.4 ± 29.3	100.8 ± 22.6	0.811
FVC (L)	1.71 ± 0.38	1.82 ± 0.30	0.019 *	2.12 ± 0.34	2.14 ± 0.36	0.627
FVC (%)	89.1 ± 22.4	98.9 ± 19.0	0.041 *	94.0 ± 21.8	99.1 ± 26.8	0.210
FEV1/FVC	0.86 ± 0.09	0.86 ± 0.10	0.889	0.89 ± 0.06	0.86 ± 0.08	0.082
MEP (cmH_2_O)	50.3 ± 18.8	48.3 ± 22.1	0.358	48.4 ± 13.5	47.0 ± 11.7	0.636
MIP (cmH_2_O)	50.3 ± 17.1	46.2 ± 18.8	0.759	52.9 ± 15.4	56.6 ± 16.8	0.243

WC: waist circumference, ASM: appendicular muscle mass, HGS: hand grip strength, 3MWD: 3-min walk distance, TC: total cholesterol, HDL-C: high-density lipoprotein cholesterol, LDL-C: low-density lipoprotein cholesterol, TG: triglycerides, FFA: free fatty acids, 25(OH)D: 25-hydroxyvitamin D, FEV1: forced expiratory volume in 1 s, FVC: functional vital capacity, MEP: maximal expiratory pressure, MIP: maximal inspiratory pressure. * *p* < 0.05.
